# IGF2BP3 promotes cell metastasis and is associated with poor patient survival in nasopharyngeal carcinoma

**DOI:** 10.1111/jcmm.17093

**Published:** 2021-12-10

**Authors:** Yun Xu, Zhoubo Guo, Hewei Peng, Lanyan Guo, Ping Wang

**Affiliations:** ^1^ Departments of Radiation Oncology Tianjin Medical University Cancer Institute and Hospital National Clinical Research Center for Cancer Key Laboratory of Cancer Prevention and Therapy Tianjin’s Clinical Research Center for Cancer Tianjin China; ^2^ Fujian Medical University Cancer Hospital Fujian Cancer Hospital Fujian China; ^3^ Department of Epidemiology and Health Statistics Fujian Provincial Key Laboratory of Environment Factors and Cancer School of Public Health Fujian Medical University Fuzhou China; ^4^ School of Medical Technology and Engineering Fujian Medical University Fuzhou China

**Keywords:** IGF2BP3, Metastasis, mTOR, Nasopharyngeal carcinoma

## Abstract

Metastasis contributes to treatment failure in nasopharyngeal carcinoma (NPC) patients. Our study aimed at elucidating the role of insulin‐like growth factor 2 mRNA binding protein 3 (IGF2BP3) in NPC metastasis and the underlying mechanism involved. IGF2BP3 expression in NPC was determined by bioinformatics, quantitative polymerase chain reaction and immunohistochemistry analyses. The biological function of IGF2BP3 was investigated by using *in vitro* and *in vivo* studies. In this study, IGF2BP3 mRNA and protein levels were elevated in NPC tissues. In addition, IGF2BP3 exerted an oncogenic role by promoting epithelial‐mesenchymal transition (EMT), thereby inducing NPC cell migration and invasion. Further studies revealed that IGF2BP3 regulated the expression of key regulators of EMT by activating AKT/mTOR signalling, thus stimulating NPC cell migration and invasion. Remarkably, targeting IGF2BP3 delayed NPC metastasis through attenuating p‐AKT and vimentin expression and inducing E‐cadherin expression *in vivo*. Moreover, IGF2BP3 protein levels positively correlated with distant metastasis after initial treatment. Importantly, IGF2BP3 expression served as an independent prognostic factor in predicting the overall survival and distant metastasis‐free survival of NPC patients. This work identifies IGF2BP3 as a novel prognostic marker and a new target for NPC treatment.

## 1 INTRODUCTION

Nasopharyngeal carcinoma (NPC) has obvious geographical distribution characteristics and is commonly diagnosed in certain regions of east and southeast Asia, such as China.^1^ In particular, NPC mainly occurs in the southern provinces of China and ranks first among head and neck malignant tumours in Fujian. Treatment with induction chemotherapy plus concurrent chemoradiotherapy has improved the 5‐year overall survival (OS) of patients with stage III‐IVB NPC to 86%, while 22% of the patients develop locoregional or distant failures.^2^ Therefore, it is necessary to clarify the underlying mechanisms of NPC metastasis and identify novel prognostic factors and targets for NPC.

Insulin‐like growth factor 2 mRNAs binding protein 3 (IGF2BP3) is a highly conserved member of the insulin‐like growth factor 2 mRNAs binding protein family. IGF2BP3 functions as a post‐transcriptional regulator that recruits target coding and non‐coding transcripts to protein‐RNA complexes and affect the expression and translation of target RNAs.^3^ IGF2BP3 was found to be involved in controlling cell invasion and metastasis, proliferation, chemoresistance and tumorigenesis.^4^, ^5^, ^6^, ^7^, ^8^, ^9^ Moreover, IGF2BP3 was also implicated in the regulation of cancer stemness, metabolism and immunity.^10^, ^11^, ^12^, ^13^ Accumulating evidence indicates that IGF2BP3 plays a tumour‐promoting role in human cancers. In renal cell carcinoma, high IGF2BP3 protein levels detected in both plasma and tissue were independently correlated with poor overall prognosis.^14^ In early invasive oesophageal adenocarcinoma, IGF2BP3 protein levels were associated with T classification and correlated with worse outcomes.^15^ However, whether IGF2BP3 plays a specific role in the progression of NPC remains undetermined.

The PI3K/AKT signalling pathway plays a critical role in regulating various cell functions, including cell survival, cell differentiation, cell growth and cell motility.^16^ Mechanistically, IGF2BP3 expression was shown to be regulated by mTOR to enhance the binding of IGF2BP3 to the 3’‐untranslated region (UTR) of the *IGF2* mRNA, thus facilitating *IGF2* expression.^17^ Furthermore, IGF2BP3 was reported to be able to activate the PI3K signalling pathway through the translational activation of IGF2 in glioblastomas and skeletal muscle satellite cells.^18^, ^19^ The regulation of IGF2 in the PI3K/AKT/mTOR signalling pathway indicated that there might be a positive feedback loop between mTOR and IGF2BP3 expression.^20^, ^21^ However, the molecular mechanisms of IGF2BP3 in NPC are largely unknown.

Here, we not only analysed the mRNA levels of IGF2BP3 in Gene Expression Omnibus (GEO) datasets but also determined the mRNA and protein levels of IGF2BP3 in clinical samples. Both mRNA and protein levels of IGF2BP3 were found to be higher in NPC than those in adjacent normal tissues. Moreover, IGF2BP3 protein expression was identified as an independent factor in predicting OS and distant metastasis‐free survival of NPC patients. Therefore, IGF2BP3 might be used to optimize therapeutic and follow‐up decisions in NPC.

## 2 MATERIALS AND METHODS

### 2.1 Bioinformatics analysis

The NPC mRNA‐Seq datasets (GSE12452, GSE13597, GSE53819, GSE61218, GDE64634, GSE126683) were downloaded from the GEO database and used to perform differential expression analysis. The differential analysis of IGF2BP3 expression was performed in NPC and adjacent normal tissue. The involvement of IGF2BP3 in specific signalling pathways was elucidated by gene set enrichment analysis (GSEA).

### 2.2 Cell lines and cell culture

Three NPC cell lines (5‐8F, HNE‐2 and C666‐1) and a nasopharyngeal epithelial cell line (NP69) were obtained from the Cell Bank of Central South University (Changsha, China) and used in this study. Cells were cultured in RPMI‐1640 medium containing 10% foetal bovine serum (FBS; Wisent Corp., Saint‐Bruno, QC, Canada) and supplemented with 1% streptomycin/penicillin (NCM Biotech, Suzhou, China) in humidified air with 5% CO_2_ at 37°C. As for the use and management of SC79, after cell transfection, cells were cultured in a complete medium with 8μg/mL SC79 for 30 mins in the incubator and then collected cells for follow‐up experiments.

### 2.3 Construction of the lentiviral vector and cell transfection

Two small hairpin RNAs (shRNAs) targeting IGF2BP3 (shIGF2BP3‐1 and shIGF2BP3‐2) were designed. A scramble shRNA, shIGF2BP3‐1 and shIGF2BP3‐2 were cloned into pLVX‐shRNA2‐puro. To construct the IGF2BP3 overexpression vector, IGF2BP3 cDNA was synthesized and cloned into pLVX‐IRES‐ZsGreen1. For lentiviral particle preparation, targeted viral plasmids were used to transfect cells with Lipofectamine 2000 (11668019; Invitrogen, Carlsbad, CA, USA). The cell lines were infected with shRNAs or overexpressed viral supernatants, and qRT‐PCR analysis and Western blot analysis were used to determine IGF2BP3 mRNA and protein expression levels, respectively.

### 2.4 Quantitative real‐time PCR analysis

Total RNA was extracted using TRIzol reagent (1596026; Invitrogen) from cells or tissue samples and stored at −80 °C in RNAse‐free H_2_O. The All‐in‐One™ First‐Strand cDNA Synthesis Kit (GeneCopoeia, Guangzhou, China) was used for reverse transcription to generate high‐quality cDNA, according to the manufacturer's protocols. A mixture containing SYBR^®^ Green Master Mix, cDNA, forward and reverse primers, RNase‐free H_2_O was prepared and subjected to qRT‐PCR. The primers used in qRT‐PCR are listed in Table S1. All experiments were performed in triplicate using glyceraldehyde‐3‐phosphate dehydrogenase (*GAPDH*) as an internal reference control. The 2^−ΔΔCT^ method was used to calculate relative mRNA levels.

### 2.5 Western blot analysis

Cell lysates from cell lines were prepared with a RIPA lysis buffer kit and were used for the quantification of protein concentrations using the bicinchoninic acid assay (Beyotime Institute of Biotechnology, Shanghai, China). After heating and denaturation, proteins were subjected to sodium dodecyl sulphate‐polyacrylamide gel electrophoresis followed by transfer to polyvinylidene fluoride membranes. After blocking with 5% skimmed milk, the membranes were individually incubated with the primary antibodies, including anti‐IGF2BP3 (1:5,000, Proteintech), anti‐E‐cadherin (1:8,000, ProteinTech Group, Chicago, IL, USA), anti‐vimentin (1:2,000, ProteinTech Group), anti‐p‐mTOR (1:1,000, Abcam, Cambridge, UK), anti‐mTOR (1:1,000, Abcam), anti‐p‐AKT (1:500, Abcam), anti‐AKT (1:500, Abcam), anti‐GAPDH (1:6,000, ProteinTech Group) and corresponding secondary antibodies. Eventually, the protein bands were detected using the enhanced chemiluminescence reagent and then scanned using the electrophoresis gel imaging analysis system. The ImageJ2 software (NIH, Bethesda, MD, USA) was used to analyse all imaging results. The density ratios of target bands to GAPDH bands were calculated and used to determine the relative protein expression.

### 2.6 Wound‐healing assay

Briefly, 5 × 10^5^ cells/well NPC cells were seeded into 24‐well plates and cultured overnight at 37°C to obtain a confluent monolayer. A 10‐µL plastic pipette tip was used to generate wounds by scratching the cell monolayer. The scathing cells were then washed with PBS and cultured in serum‐free RPMI‐1640. An inverted microscope was used for recording the wounds at time points of 0 and 24 h. Ultimately, the ImageJ (version 1.53a) was used to measure the width of wound‐healing assays. After measurement of the grey value of wounds, the formula (width of wound = (grey value*500)/255) was used to calculate the width.

### 2.7 Transwell assay

Transwell chambers (Corning Inc., Corning, NY, USA) with or without Matrigel (BD Biosciences, San Jose, CA, USA) were used for Transwell invasion and migration assays. Briefly, cell suspension was prepared in serum‐free RPMI‐1640, and 1 × 10^5^ cells/well in 100 μL serum‐free RPMI‐1640 was added to the upper wells of the chambers, and RPMI‐1640 with 10% FBS was added to the lower wells of the chambers. After 30 h for the migration assays or 48 h for the invasion assays, the cells that passed through the chambers were fixed with 4% paraformaldehyde and were then stained with 0.5% crystal violet followed by counting under a microscope.

## 3 NUDE MICE IN VIVO EXPERIMENTS

The 4‐ to 6‐week‐old male Balb/c athymic nude mice used in this study were obtained from the Shanghai Laboratory Animal Company (SLAC), Ltd. (Shanghai, China). All mice were housed in a specific‐pathogen‐free animal facility. All animal experiment protocols used in this work were approved by the Ethics Committee for Animal Experiments of the Fujian Medical University Cancer Hospital, and experiments were conducted according to the Guide for the Care and Use of Laboratory Animals. For the lung metastasis model, cell lines assigned to the shNC, shIGF2BP3‐1, and shIGF2BP3‐2 groups were inoculated into nude mice through the tail vein (200 µL cell suspension, 4 × 10^6^ cells per mouse). After 3–4 weeks of cell inoculation, the mice were euthanized and the number of lung surface metastatic foci was counted. The lung tissues were also harvested for immunohistochemistry (IHC) staining and Western blot analysis.

### 3.1 Collection of clinical samples

We selected 112 patients with NPC between 2007 and 2010. The inclusion criteria were as follows: (1) histologically confirmed NPC; (2) with complete follow‐up data; (3) with enough tissue section. The exclusion criteria were as follows: NPC patients who received treatments before a biopsy, such as chemotherapy, radiotherapy, molecular targeted therapy or immunotherapy. All patients underwent a biopsy and had a definitive pathological diagnosis. The medical records of the patients were used for clinical data extraction. All the patients provided written informed consent before sample collection, and the Ethics Committee of the hospital approved the protocols, the ethics approval code was K2021‐092‐01.

### 3.2 Immunohistochemistry analysis

The immunohistochemistry (IHC) analysis was performed on 4‐µm paraffin‐embedded NPC tissue sections. After deparaffinized in xylene and rehydrating in alcohol, the sections were boiled in antigen retrieval buffers and immersed with 3% hydrogen peroxide to block endogenous peroxidase activity. The sections were treated with normal goat serum followed by individual incubation with the appropriate primary antibody against IGF2BP3 (1:200, ProteinTech Group), E‐cadherin (1:800, ProteinTech Group), vimentin (1:3,000, ProteinTech Group), p‐AKT antibody (1:100, Abcam) and AKT (1:800, Abcam). After incubation with corresponding secondary antibodies, the sections were counterstained with Mayer's haematoxylin to stain the nucleus and were then dehydrated and mounted for imaging. The images of the slides were captured under a microscope. Two independent pathologists blindly evaluated the IHC scoring of these slides with software assistance. For IGF2BP3 evaluation, the intensity scores were designated as 0 (no), 1 (weak), 2 (moderate) and 3 (strong) and the percentages of the stained cells were divided into five levels as follows: 0 (<5%), 1 (5–25%), 2 (26–50%), 3 (51–75%) and 4 (76–100%) and used to record the percentage score. The final score was calculated by multiplying the intensity score and percentage score. A score >6 was defined as high expression, while a score ≤6 was defined as low expression.

### 3.3 Statistical analysis

Statistical analyses were conducted using the SPSS (version 21.0) software (IBM Corp., Armonk, NY, USA) and GraphPad Prism (version 9.0.0) software (GraphPad Software Inc., San Diego, CA, USA). The experimental results were expressed as the mean ±standard deviation (SD). Wilcoxon rank‐sum tests were adopted for evaluating the differential IGF2BP3 expression between NPC and normal tissues. The χ^2^ test was performed to elucidate the association between IGF2BP3 expression and clinical parameters of NPC. Survival analyses were performed by plotting Kaplan‐Meier survival curves, and log‐rank tests were performed to evaluate the difference. The univariate and multivariate Cox proportional hazards models were used to evaluate the effects of various variables on NPC patient survival, and only the significant factors identified in univariate analyses were further used for multivariate analyses. As for the *in vitro* and *in vivo* experimental results, the differences between groups were assessed by one‐way analysis of variance (ANOVA) and two‐tailed t tests. All statistical tests were two‐sided and a *P* value of <0.05 represented statistical significance.

## 4 RESULTS

### 4.1 IGF2BP3 expression levels are increased in NPC tissues and cells

**FIGURE 1:**
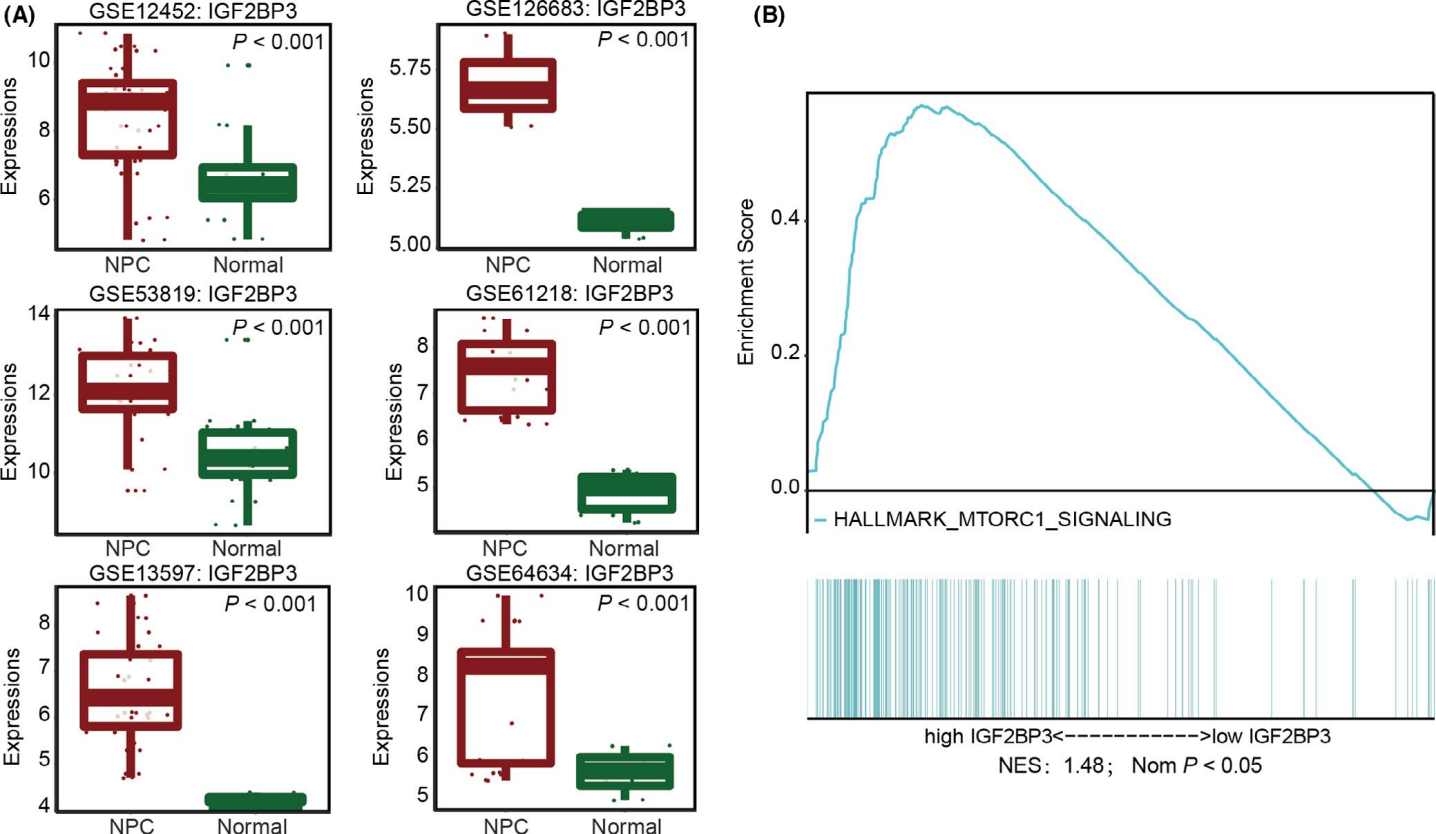
IGF2BP3 is increased in nasopharyngeal carcinoma based on GEO datasets. (A) The differential expression of IGF2BP3 mRNA levels between nasopharyngeal carcinoma and adjacent normal tissues in the GEO database (GSE12452, GSE13597, GSE53819, GSE61218, GDE64634, GSE126683). (B) Gene set enrichment analysis (GSEA) indicated that IGF2BP3 is positively involved in mTORC1 signalling. Wilcoxon rank‐sum tests were used to evaluate the differential expression levels of IGF2BP3 between NPC and normal tissues

The differential gene expression analyses were performed using The Cancer Genome Atlas (TCGA) database and suggested that the IGF2BP3 mRNA levels were elevated in most human cancers compared to the corresponding normal tissues based on the pan‐cancer view of IGF2BP3 in human cancers, including head and neck cancer (http://gepia.cancer‐pku.cn/). To further characterize the role of IGF2BP3 in NPC, we performed bioinformatic analysis of the GEO datasets and determined that the IGF2BP3 mRNA levels were differentially expressed between NPC and adjacent normal tissues (*p *< 0.001) (Figure 1A). Also, the examination of the role of IGF2BP3 in NPC by GSEA revealed that IGF2BP3 was positively involved in mTORC1 signalling (Figure 1B).

**FIGURE 2:**
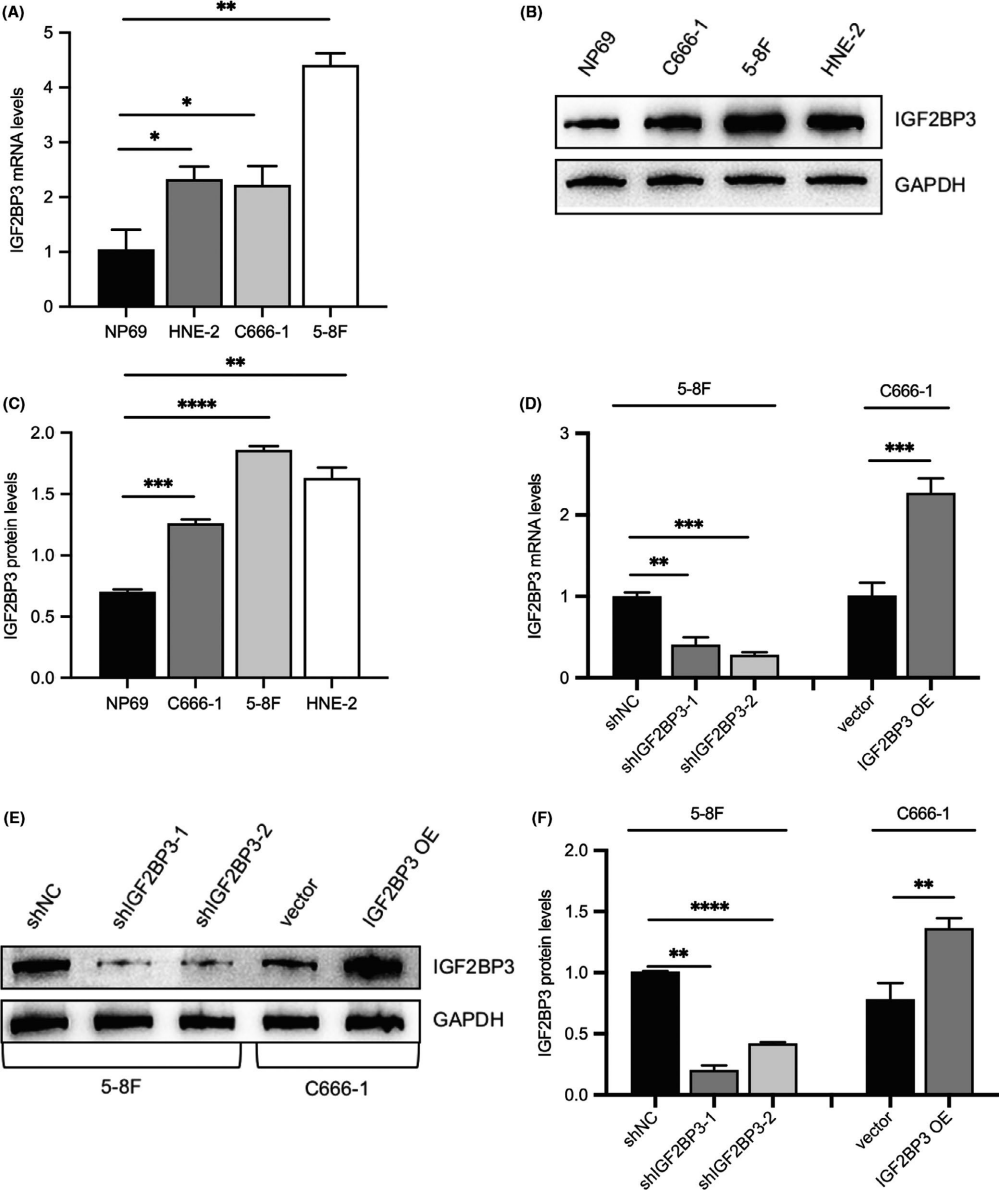
The detection of IGF2BP3 expression levels in nasopharyngeal epithelial cell line and nasopharyngeal carcinoma cell lines. (A) IGF2BP3 mRNA expression in NP69, HNE‐2, C666‐1 and 5‐8F cell lines. (B, C) IGF2BP3 protein expression in NP69, HNE‐2, C666‐1 and 5‐8F cell lines. (D–F) qRT‐PCR and Western blot analyses were performed to confirm the silencing and overexpression efficiency of IGF2BP3 in 5‐8F and C666‐1. Differences between groups were evaluated using two‐tailed t tests or one‐way analysis of variance (ANOVA). (*p < 0.05, **p < 0.01, ***p < 0.001, ****p < 0.0001)

In addition, the mRNA and protein levels of IGF2BP3 were measured in 5‐8F, C666‐1, HNE‐2 and NP69 to ascertain whether IGF2BP3 overexpression is a characteristic of NPC cell lines. As shown in Figure 2A–C, IGF2BP3 expression in NPC Cell Lines was higher than that in nasopharyngeal epithelial cell line. The effects of silencing the expression of IGF2BP3 were studied in 5‐8F cells with relatively high IGF2BP3 expression infected with lenti‐shIGF2BP3 lentivirus and control lentivirus, and C666‐1 cells with relatively weak IGF2BP3 expression infected with GFP‐tagged IGF2BP3 lentivirus and control lentivirus. The silencing and overexpression efficiency were confirmed by qRT‐PCR and Western blot analyses. Compared to the control group, IGF2BP3 expression was decreased by shIGF2BP3‐1 (40%) and shIGF2BP3‐2 (28%) in 5‐8F cells and was increased in IGF2BP3‐overexpressing C666‐1 cells (Figure 2D–F).

## 5 SILENCING OF IGF2BP3 INHIBITED NPC CELL MIGRATION AND INVASION IN VIVO AND IN VITRO

**FIGURE 3:**
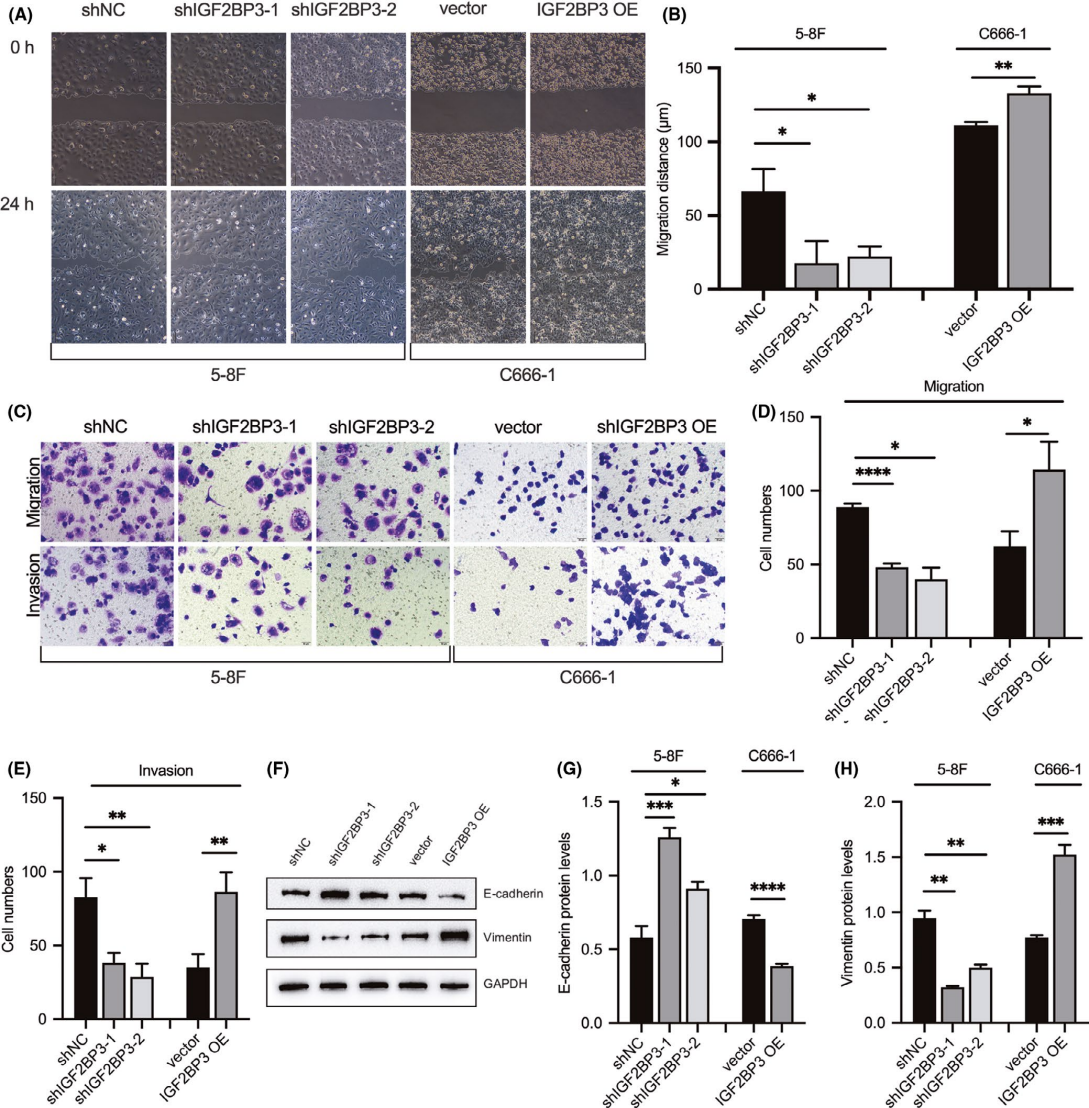
Effects of IGF2BP3 silencing and overexpression on nasopharyngeal carcinoma cell migration and invasion. (A, B) Wound‐healing assays were used to determine the migration ability of IGF2BP3‐silenced 5‐8F cells, IGF2BP3‐overexpressing C666‐1, and their corresponding controls. (C–E) Transwell assays were performed to evaluate the migration and invasion ability of IGF2BP3‐silenced 5‐8F cells, IGF2BP3‐overexpressing C666‐1, and their corresponding controls. (F–H) E‐cadherin and vimentin protein expression in IGF2BP3‐silenced 5‐8F cells, IGF2BP3‐overexpressing C666‐1, and their corresponding controls. All cell images were magnified 400 times. Differences between groups were assessed using two‐tailed t tests or one‐way analysis of variance (ANOVA). (*p < 0.05, **p < 0.01, ***p < 0.001, ****p < 0.0001)

**FIGURE 4:**
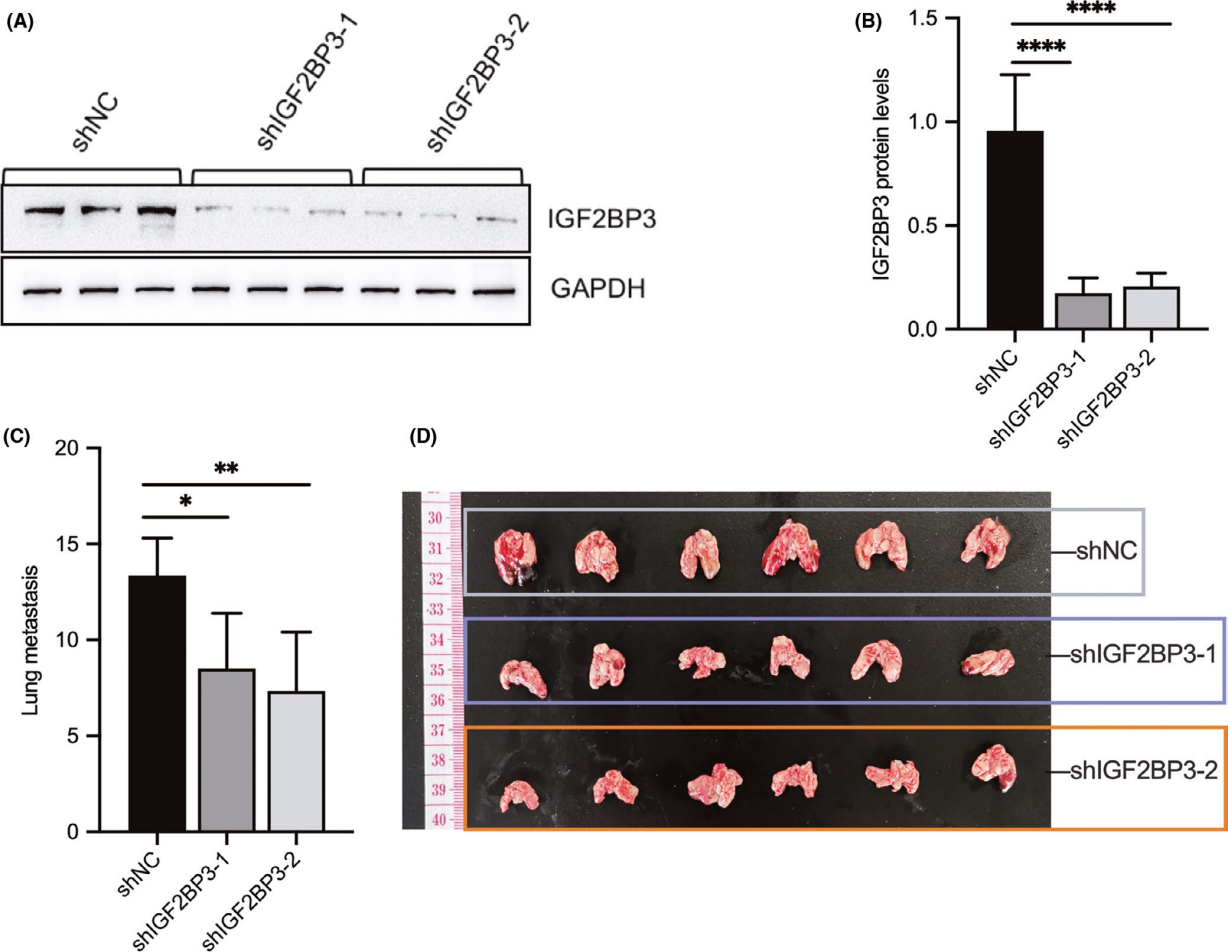
Knockdown of IGF2BP3 delays nasopharyngeal carcinoma cell metastasis in vivo. (A, B) IGF2BP3 protein expression was determined by Western blot analysis in the IGF2BP3‐silenced xenografts and their controls. (C, D) Knockdown of IGF2BP3 reduced the number of lung metastasis in xenografts. All cell images were magnified 400 times. Differences between groups were assessed using two‐tailed t tests or one‐way analysis of variance (ANOVA). (*p < 0.05, **p < 0.01, ***p < 0.001, ****p < 0.0001)

The evaluation of the role of IGF2BP3 in the migration and invasion of NPC cells by the wound‐healing and transwell assays revealed that IGF2BP3 knockdown could impair the wound‐healing rate of 5‐8F cells. In contrast, the ectopic overexpression of IGF2BP3 in C666‐1 cells had the opposite effect (Figure 3A and B). Consistently, silencing of IGF2BP3 expression inhibited the migrating and invading cells passing through the membrane with or without Matrigel, whereas overexpression of IGF2BP3 had the opposite effect (Figure 3C–E). Moreover, the evaluation of the effects of IGF2BP3 on representative EMT markers, including vimentin and E‐cadherin, in NPC cells by Western blot analysis showed upregulation of E‐cadherin with IGF2BP3 knockdown and downregulation of vimentin with IGF2BP3 depletion. However, overexpression of IGE2BP3 had the opposite effects (Figure 3F–H). The evaluation of the role of IGF2BP3 in NPC metastasis in our established lung metastasis model confirmed the downregulation of IGF2BP3 expression levels in the xenografts (Figure 4A and B). Compared with those in the control group, mice injected with IGF2BP3‐silenced NPC cells developed fewer lung metastases (Figure 4C and D).

### 5.1 IGF2BP3 knockdown inhibited NPC cell migration and invasion through AKT/mTORC1 signalling

**FIGURE 5:**
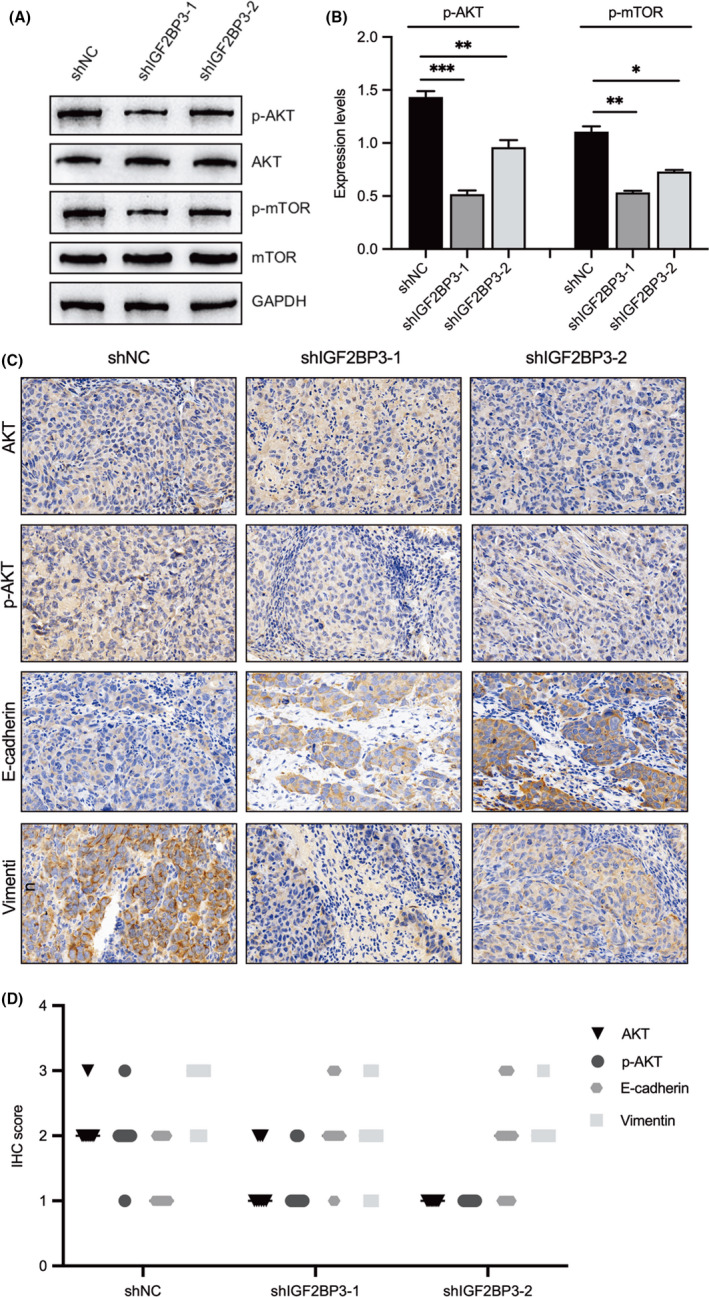
Silencing of IGF2BP3 impairs AKT/mTOR signalling. (A, B) Western blot analysis was performed to evaluate the effect of IGF2BP3 knockdown on the protein expression of p‐AKT, AKT, p‐mTOR and mTOR. (C, D) Immunohistochemistry (IHC) staining was used to detect AKT, p‐AKT, E‐cadherin and vimentin expression in xenografts. All cell images were magnified 400 times. Differences between groups were assessed using two‐tailed *t* tests or one‐way analysis of variance (ANOVA). (*p < 0.05, **p < 0.01, ***p < 0.001, ****p < 0.0001)

**FIGURE 6:**
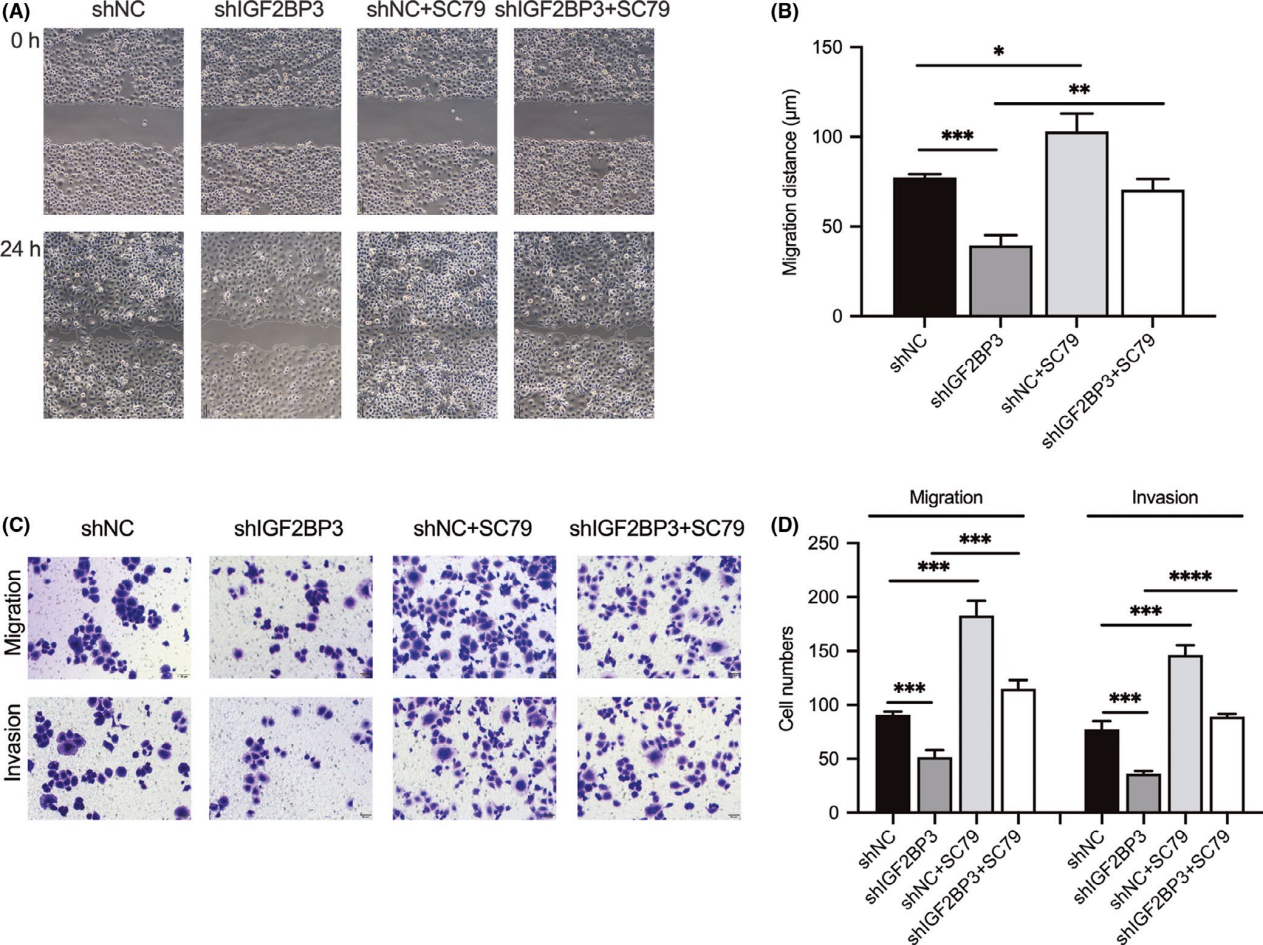
Silencing of IGF2BP3 impairs nasopharyngeal carcinoma cell migration and invasion through AKT/mTOR signalling. (A, B) Wound‐healing assays were performed to evaluate the migration ability of IGF2BP3‐silenced 5‐8F cells, SC79‐treated C666‐1, IGF2BP3‐silenced 5‐8F cells with SC79 treatment and their controls. (C, D) Transwell assays were performed to evaluate the migration and invasion ability of IGF2BP3‐silenced 5‐8F cells, SC79‐treated 5‐8F, IGF2BP3‐silenced 5‐8F cells with SC79 treatment and their controls. Differences between two groups were assessed using two‐tailed t tests. (*p < 0.05, **p < 0.01, ***p < 0.001, ****p < 0.0001)

The GSEA analysis revealed that IGF2BP3 expression was associated with the mTORC1 signalling pathway, which is well known to be involved in modulating cell migration and invasion. The evaluation of the effect of IGF2BP3 on AKT/mTOR signalling in NPC cells by Western blot analysis indicated that silencing of IGF2BP3 reduced the phosphorylation of AKT and mTOR (Figure 5A and B). Moreover, the results of the IHC analysis of xenografts consistently showed that IGF2BP3 depletion downregulated p‐AKT and vimentin and upregulated E‐cadherin (Figure 5C and D). Furthermore, the association between IGF2BP3 and AKT/mTOR signalling was verified by treating the IGF2BP3‐silenced NPC cells with an AKT signalling activator, SC79. The results showed that compared with the controls, the activation of AKT signalling promoted NPC cell migration and invasion and reversed the inhibitory effect induced by knockdown of IGF2BP3 (Figure 6). Collectively, these results indicate that IGF2BP3 functions as an oncogene possibly by activating the AKT/mTOR signalling pathway in NPC cells.

### 5.2 The association between IGF2BP3 protein levels and clinicopathological parameters of NPC patients

**FIGURE 7:**
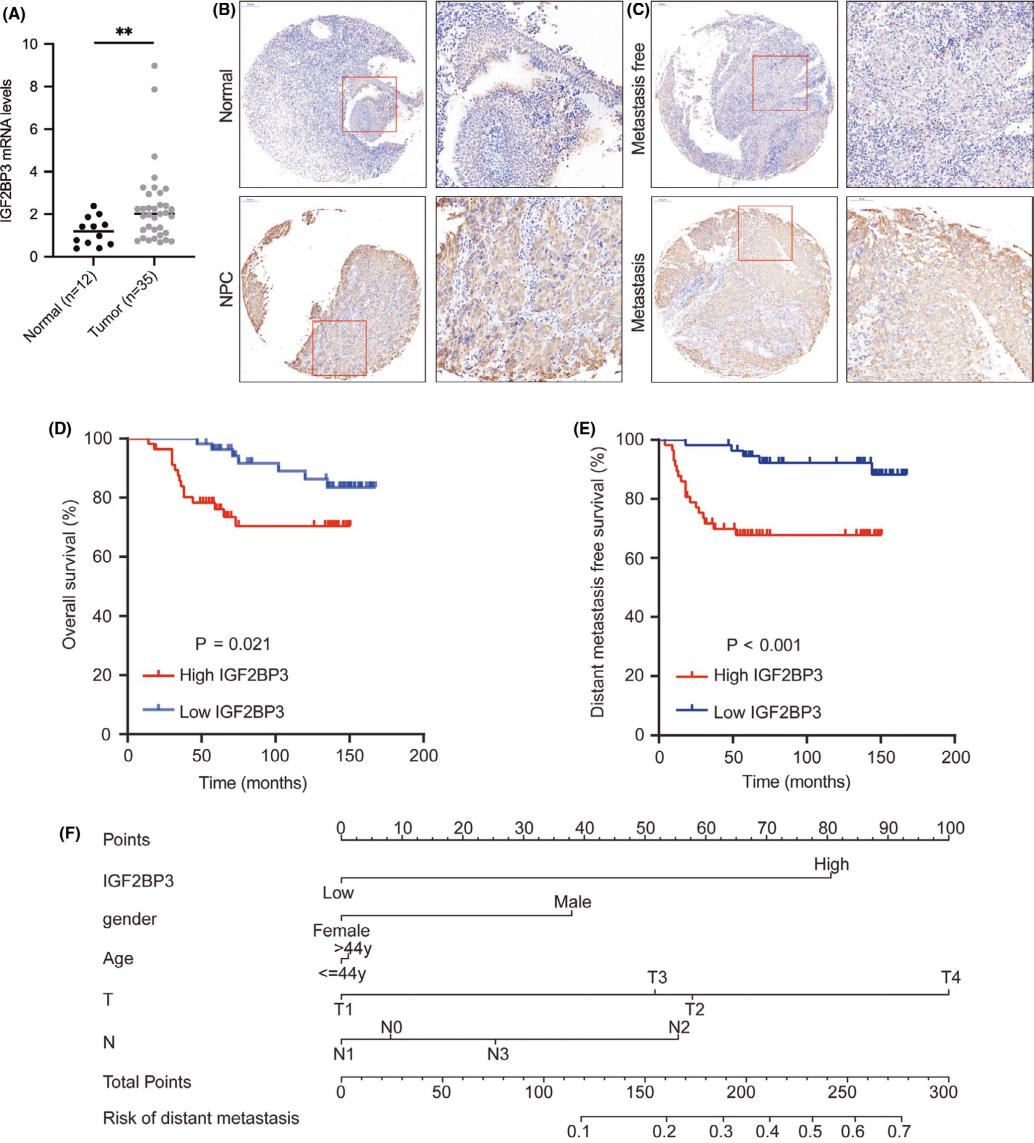
IGF2BP3 mRNA and protein levels in nasopharyngeal carcinoma and the relationship between IGF2BP3 expression and patient prognosis. (A) qRT‐PCR analysis was performed to determine IGF2BP3 mRNA levels in 35 fresh nasopharyngeal carcinoma and 12 adjacent normal tissues. (B) Immunohistochemistry (IHC) staining was performed to measure IGF2BP3 protein levels in 112 paraffin‐embedded nasopharyngeal carcinoma and 32 paraffin‐embedded adjacent normal tissues. (C) IGF2BP3 protein levels were positively correlated with distant metastasis after initial treatment. (D, E) High IGF2BP3 expression was associated with poor overall survival and distant metastasis‐free survival. (F) The nomogram of IGF2BP3 and other clinicopathological characteristics in predicting distant metastasis. Expression differences between NPC and normal tissue were assessed using Wilcoxon rank‐sum tests. Survival analyses were performed by plotting Kaplan‐Meier survival curves, and a log‐rank test was used to evaluate the difference. (*p < 0.05, **p < 0.01, ***p < 0.001, ****p < 0.0001)

Consistent with the results obtained by bioinformatics analysis, the results of the determination of the IGF2BP3 expression levels by qRT‐PCR and IHC analyses revealed upregulated mRNA and protein expression levels of IGF2BP3 in NPC tissues (Figure 7 A and B) (Table S2). According to the clinicopathological parameters of 112 NPC patients, shown in Table S3, the median age of the NPC patients was 44 years old, and 83 patients (74.1%) were male. The correlation analyses revealed a positive correlation between IGF2BP3 protein levels and distant metastasis after initial treatment (Figure 7C). However, no correlation was found between IGF2BP3 protein levels and other clinicopathological parameters, such as age, sex, clinical T classification, clinical N classification, clinical stage and recurrence after initial treatment in NPC patients (Table S3).

Survival analyses to determine the association between IGF2BP3 protein levels and the OS of NPC patients suggested a positive association between high IGF2BP3 protein levels and poor OS of NPC patients (Figure 7D). Given that IGF2BP3 expression was associated with distant metastasis after initial treatment, we investigated the connection between IGF2BP3 expression and the DMFS of NPC patients. Consistently, survival analyses showed that high IGF2BP3 protein levels were associated with a poor DMFS in NPC patients (Figure 7E). Univariate and multivariate analyses were performed to probe the link between IGF2BP3 protein levels and the clinicopathological parameters and OS and DMFS of the NPC patients. Multivariate Cox proportional hazard analyses identified IGF2BP3 expression as an independent prognostic factor for the OS and distant metastasis‐free survival of NPC patients (Table S4 and Table S5). Moreover, age was also identified as an independent prognostic factor for the OS of NPC patients. Furthermore, the nomogram showed the role of IGF2BP3 and other clinicopathological characteristics in predicting cancer metastasis in NPC patients after initial treatment (Figure 7F).

## 6 DISCUSSION

IGF2BP3 acts as an oncogene in various human cancers.^3^ However, the function of IGF2BP3 in NPC remains unclear. In this study, we investigated the role of IGF2BP3 in NPC cell metastasis. Bioinformatics analysis in combination with IHC analysis of clinical samples revealed that IGF2BP3 has an oncogenic role in NPC.

Both IGF2BP3 mRNA and protein levels were found to be upregulated in cutaneous squamous cell carcinoma.^22^ Moreover, IGF2BP3 protein levels were shown to be specifically expressed in intrahepatic cholangiocarcinoma compared to the surrounding normal tissue.^23^ Through bioinformatics, qRT‐PCR and IHC analyses, this study also found that both mRNA and protein levels of IGF2BP3 were higher in NPC than those in adjacent normal tissues. Moreover, we also confirmed the elevated expression of IGF2BP3 in NPC cells.

IGF2BP3 was previously demonstrated to be an upstream regulator of AKT/mTOR signalling in hepatocellular carcinoma.^24^ In addition, AKT/mTOR signalling was shown to be responsible for the EMT process, thereby promoting NPC cell migration and invasion.^25^ In this study, GSEA also revealed the involvement of IGF2BP3 in the regulation of mTOR signalling. Additionally, we confirmed that IGF2BP3 promotes NPC cell migration and invasion. Further investigation revealed that IGF2BP3 induces EMT in NPC cells through AKT/mTOR signalling, thus promoting NPC metastasis. Moreover, the results of the IHC analysis of xenografts confirmed the role of IGF2BP3 in inducing the EMT process through AKT signalling, indicating that IGF2BP3 functions as an oncogene in NPC cells by activating the AKT/mTOR signalling pathway.

IGF2BP3 expression was also shown to be correlated with the clinical stage in urachal carcinoma and ovarian clear cell carcinoma^26^, ^27^ and associated with lymphatic invasion and histological grade,^28^ lesion depth^29^ and poor OS and disease‐free survival.^30^, ^31^ This study only found a positive relationship between IGF2BP3 expression and distant metastasis after initial treatment in NPC. Furthermore, this study showed the negative link between OS, DMFS and IGF2BP3 protein levels. Notably, IGF2BP3 expression was demonstrated to be an independent factor in evaluating the prognosis of NPC patients. We also established a nomogram for predicting cancer metastasis after initial treatment of NPC patients and for the decision‐making process of clinicians. To this end, we proposed that the abnormal expression of IGF2BP3 can be used as a key indicator of the pathogenesis of NPC.

Overall, we proposed that IGF2BP3 functions as a promoter of metastasis in NPC. IGF2BP3 protein levels are positively correlated with distant metastasis after initial treatment and negatively related with OS and DMFS in patients with NPC. IGF2BP3 may be used as a new target for the treatment of NPC.

## CONFLICT OF INTEREST

The authors confirm that there are no conflicts of interest.

## AUTHOR CONTRIBUTION

**Yun Xu:** Conceptualization (equal); Formal analysis (equal); Funding acquisition (equal); Investigation (equal); Writing – original draft (equal). **Zhoubo Guo:** Formal analysis (equal); Investigation (equal); Writing – original draft (equal). **Hewei Peng:** Investigation (equal); Resources (equal); Supervision (equal). **Lanyan Guo:** Formal analysis (equal); Resources (equal); Supervision (equal). **Ping Wang:** Conceptualization (equal); Funding acquisition (equal); Project administration (lead).

## Supporting information

Table S1‐S5

## Data Availability

The data that support the findings of this study are available from the corresponding author upon reasonable request.
