# Correction to IGF2BP3 promotes cell metastasis and is associated with poor patient survival in nasopharyngeal carcinoma

**DOI:** 10.1111/jcmm.18036

**Published:** 2023-12-11

Xu Y, Guo Z, Peng H, Guo L, Wang P. IGF2BP3 promotes cell metastasis and is associated with poor patient survival in nasopharyngeal carcinoma. *J Cell Mol Med.* 2022;26(2):410–421. doi:10.1111/jcmm.17093

In Yun Xu et al., the bands corresponding to p‐AKT and p‐mTOR in Figure 5A on page 417 were inadvertently duplicated during the figure preparation for publication. The accurate image is provided below. It is important to note that this error does not impact any of the statistical analyses or conclusions drawn in the article. The authors confirm that all results and conclusions presented in this paper remain unchanged.

**FIGURE 5:**
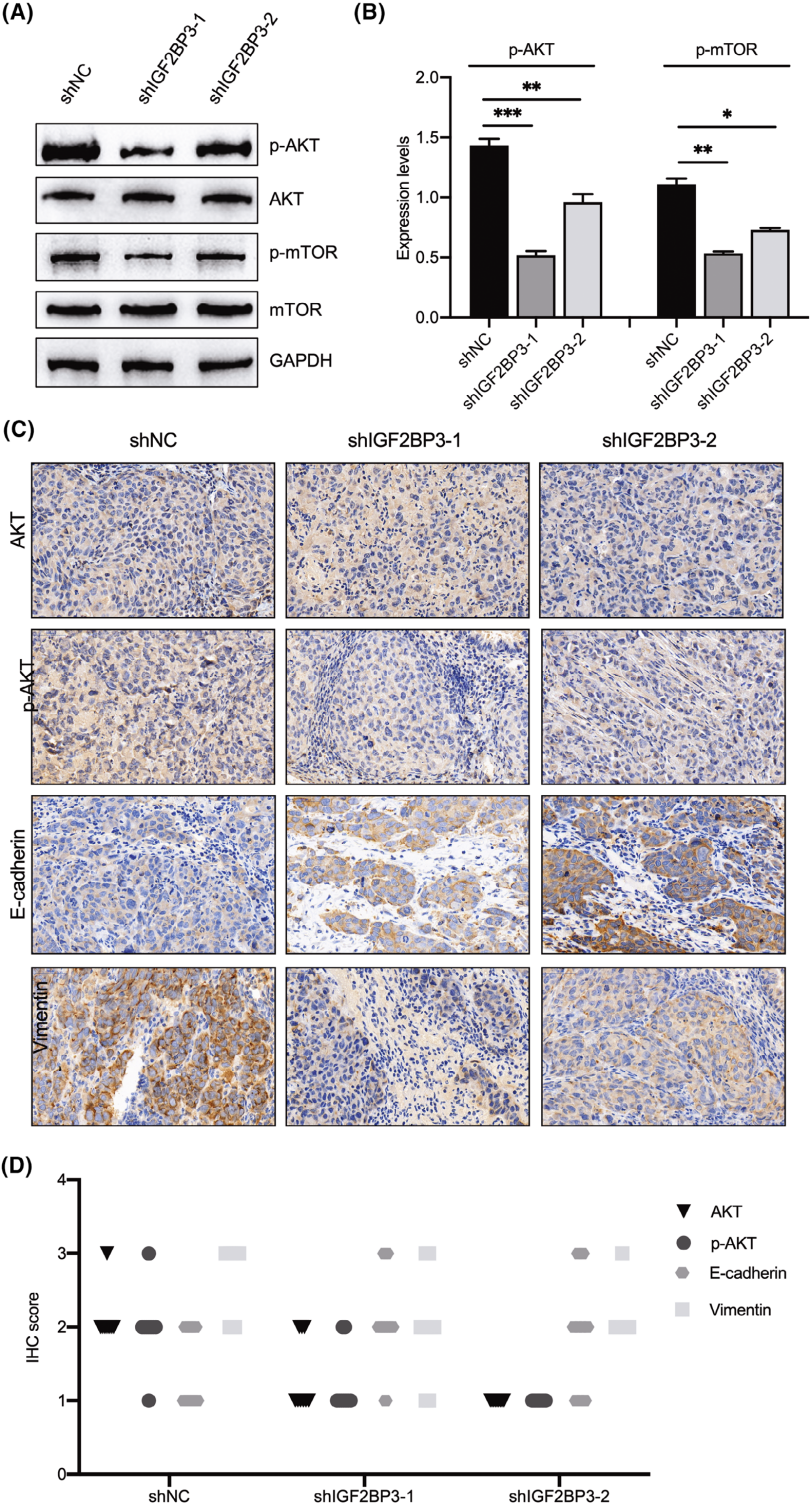
Silencing of IGF2BP3 impairs AKT/mTOR signalling. (A, B) Western blot analysis was performed to evaluate the effect of IGF2BP3 knockdown on the protein expression of p‐AKT, AKT, p‐mTOR and mTOR. (C, D) Immunohistochemistry (IHC) staining was used to detect AKT, p‐AKT, E‐cadherin and vimentin expression in xenografts. All cell images were magnified 400 times. Differences between groups were assessed using two‐tailed *t* tests or one‐way analysis of variance (anova). (*p < 0.05, **p < 0.01, ***p < 0.001, ****p < 0.0001).

