# Supplementary material for: IGF2BP3 promotes cell metastasis and is associated with poor patient survival in nasopharyngeal carcinoma
Source: J Cell Mol Med. 2021 Dec 10;26(2):410–21. doi: 10.1111/jcmm.17093 (PMC8743660; doi:10.1111/jcmm.17093)
Supplement: Supplementary file 1 — Table S1‐S5 [file JCMM-26-410-s001.doc]

Supplementary Tables

**Table S1. Primers used for quantitative real-time PCR (qRT-PCR)**

| Genes | Forward primer (5’-3’) | Reverse primer (5’-3’) |
| --- | --- | --- |
| GAPDH  IGF2BP3 | ACAGCCTCAAGATCATCAGC  TTGGAAAAGGAGGCAAAACG | GGTCATGAGTCCTTCCACGAT  GGCAACCTGGCAAGCATAGA |

| **Table S2. IGF2BP3 protein levels in nasopharyngeal carcinoma and adjacent normal tissues** | | | | |
| --- | --- | --- | --- | --- |
| Group | Cases (n) | IGF2BP3 protein levels | | *P* value |
|  |  | Low | High |  |
| Adjacent normal tissues | 32 | 25 (78.1%) | 7 (21.9%) | 0.004 |
| Nasopharyngeal carcinoma | 112 | 55 (49.1%) | 57 (50.9%) |

| **Table S3. Relationship between IGF2BP3 protein levels and the clinicopathological characteristics of nasopharyngeal carcinoma patients** | | | | |
| --- | --- | --- | --- | --- |
| Characteristics | Total | IGF2BP3 protein levels | | *P* value |
|  |  | Low, n (%) | High, n (%) |  |
| Age (years) |  |  |  |  |
| ≤Median | 66 | 35 (53.0%) | 31 (47.0%) | 0.320 |
| ＞Median | 46 | 20 (43.5%) | 26 (56.5%) |  |
| Sex |  |  |  |  |
| Male | 83 | 40 (48.2%) | 43 (51.8%) | 0.743 |
| Female | 29 | 15 (51.7%) | 14 (48.3%) |  |
| cT stage |  |  |  |  |
| T1 | 14 | 7 (50.0%) | 7 (50.0%) | 0.484 |
| T2 | 22 | 10 (45.5%) | 12 (54.5%) |  |
| T3 | 54 | 30 (55.6%) | 24 (44.4%) |  |
| T4 | 22 | 8 (36.4%) | 14 (63.6%) |  |
| cN stage |  |  |  |  |
| N0 | 17 | 9 (52.9%) | 8 (47.1%) | 0.796 |
| N1 | 56 | 28 (50.0%) | 28 (50.0%) |  |
| N2 | 30 | 15 (50.0%) | 15 (50.0%) |  |
| N3 | 9 | 3 (33.3%) | 6 (66.7%) |  |
| Clinical stage |  |  |  |  |
| I | 4 | 3 (75.0%) | 1 (25.0%) | 0.189 |
| II | 19 | 10 (52.6%) | 9 (47.4%) |  |
| III | 59 | 32 (54.2%) | 27 (45.8%) |  |
| IV | 30 | 10 (33.3%) | 20 (66.7%) |  |
| Recurrence after initial treatment |  |  |  |  |
| No | 103 | 52 (50.5%) | 51 (49.5%) | 0.324 |
| Yes | 9 | 3 (33.3%) | 6 (66.7%) |  |
| Metastasis after initial treatment |  |  |  |  |
| No | 89 | 50 (56.2%) | 39 (43.8%) | 0.003 |
| Yes | 23 | 5 (21.7%) | 18 (78.3%) |  |

| **Table S4. Univariate and multivariate survival analyses of clinicopathological variables of nasopharyngeal carcinoma patients** | | | | | | | |
| --- | --- | --- | --- | --- | --- | --- | --- |
|
|  | Overall survival | | | | | | |
|  | Univariate analysis | | |  | Multivariate analysis | | |
| Clinical characteristics | HR | 95% CI | *P value* |  | HR | 95% CI | *P value* |
| IGF2BP3 protein levels | 2.777 | (1.128-6.836) | 0.021 |  | 2.724 | (1.078-6.884) | 0.034 |
| High *vs.* Low |  |  |  |  |  |  |  |
| Age (years) | 3.039 | (1.270-7.271) | 0.013 |  | 2.937 | (1.217-7.088) | 0.016 |
| ＞Median *vs.* ≤Median |  |  |  |  |  |  |  |
| Sex | 0.419 | (0.124-1.415) | 0.161 |  |  |  |  |
| Female *vs.* Male |  |  |  |  |  |  |  |
| cT stage | 1.246 | (0.755-2.056) | 0.390 |  |  |  |  |
| T3-T4 *vs.* T1-T2 |  |  |  |  |  |  |  |
| cN stage | 1.139 | (0.674-1.926) | 0.627 |  |  |  |  |
| N1-3 *vs.* N0 |  |  |  |  |  |  |  |

| **Table S5. Univariate and multivariate survival analyses of clinicopathological variables of nasopharyngeal carcinoma patients** | | | | | | | |
| --- | --- | --- | --- | --- | --- | --- | --- |
|
|  | Distant metastasis-free survival | | | | | | |
|  | Univariate analysis | | |  | Multivariate analysis | | |
| Clinical characteristics | HR | 95% CI | *P value* |  | HR | 95% CI | *P value* |
| IGF2BP3 protein levels | 4.578 | (1.667-12.576) | < 0.001 |  | 4.578 | (1.667-12.576) | 0.003 |
| High *vs.* Low |  |  |  |  |  |  |  |
| Age (years) | 1.408 | (0.621-3.194) | 0.413 |  |  |  |  |
| ＞Median *vs.* ≤Median |  |  |  |  |  |  |  |
| Sex | 0.121 | (0.161-1.371) | 0.147 |  |  |  |  |
| Female *vs.* Male |  |  |  |  |  |  |  |
| cT stage | 1.617 | (0.958-2.728) | 0.072 |  |  |  |  |
| T3-T4 *vs.* T1-T2 |  |  |  |  |  |  |  |
| cN stage | 1.280 | (0.783-2.095) | 0.325 |  |  |  |  |
| N1-3 *vs.* N0 |  |  |  |  |  |  |  |
